# Supplementary material for: Incomplete human reference genomes can drive false sex biases and expose patient-identifying information in metagenomic data
Source: Nat Commun. 2025 Jan 18;16:825. doi: 10.1038/s41467-025-56077-5 (PMC11742726; doi:10.1038/s41467-025-56077-5)
Supplement: Supplementary file 1 — Supplementary Information [file 41467_2025_56077_MOESM1_ESM.pdf]

Supplementary Information

Incomplete human reference genomes can drive false sex biases and expose patient-identifying information in metagenomic data

Supplementary Figures 1-5

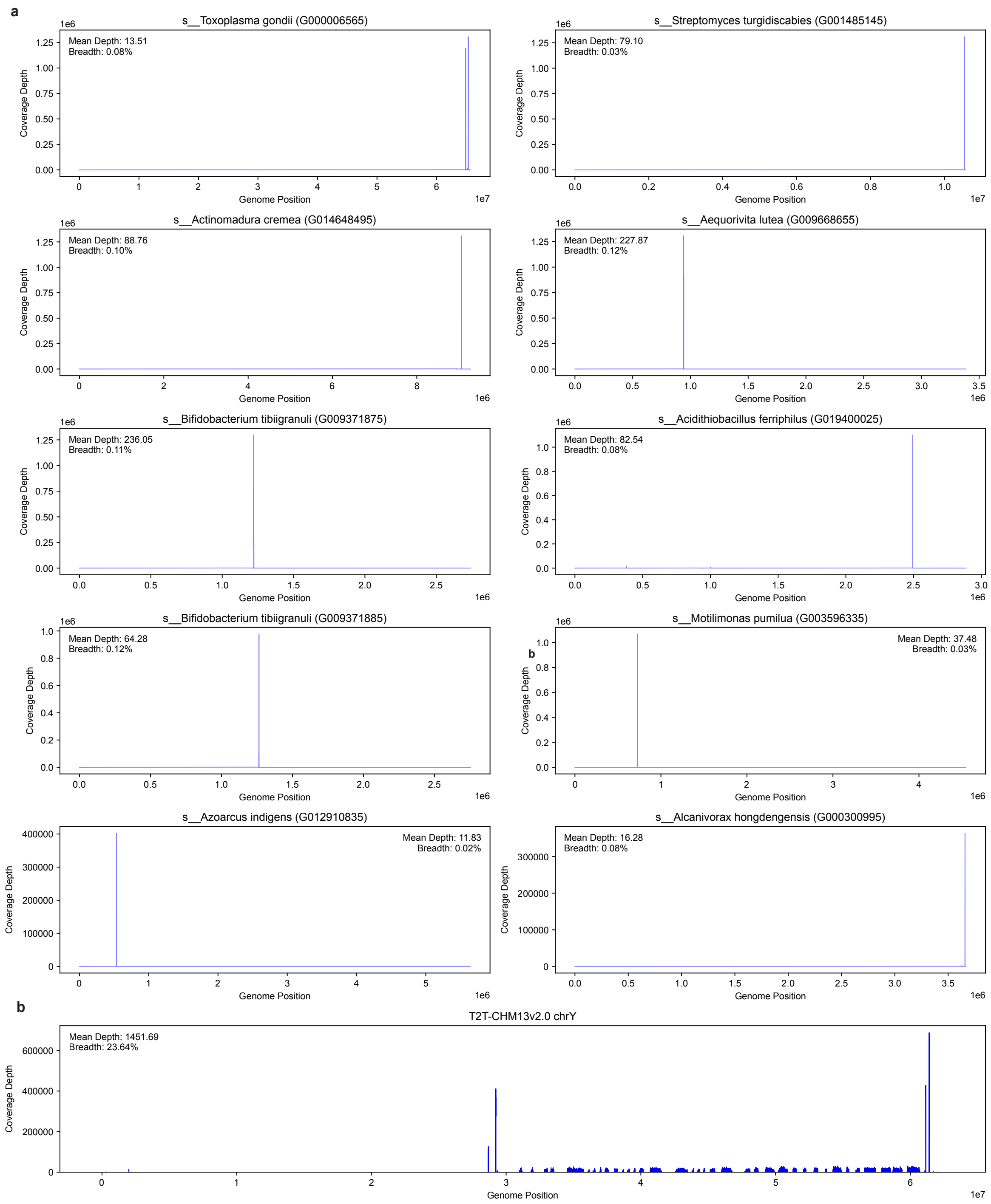

Supplementary Figure 1. Coverage depth and breadth distribution for top mismapped genomes from RefSeq210 (a) Filtered reads from the 100 HMF colorectal tumor tissue samples removed following inclusion of the T2T-CHM13v2.0 human reference genome (Method 1; step ii) were aggregated. Coverage depth and breadth are reported for the top 10 resulting microbial taxa, annotated by species and OGU identifier, following alignment of the aggregated reads to RefSeq210 via the SHOGUN parameter set. (b) Coverage depth and breadth for the same filtered and aggregated reads from (a) aligned to the Y-chromosome of T2T-CHM13v2.0.

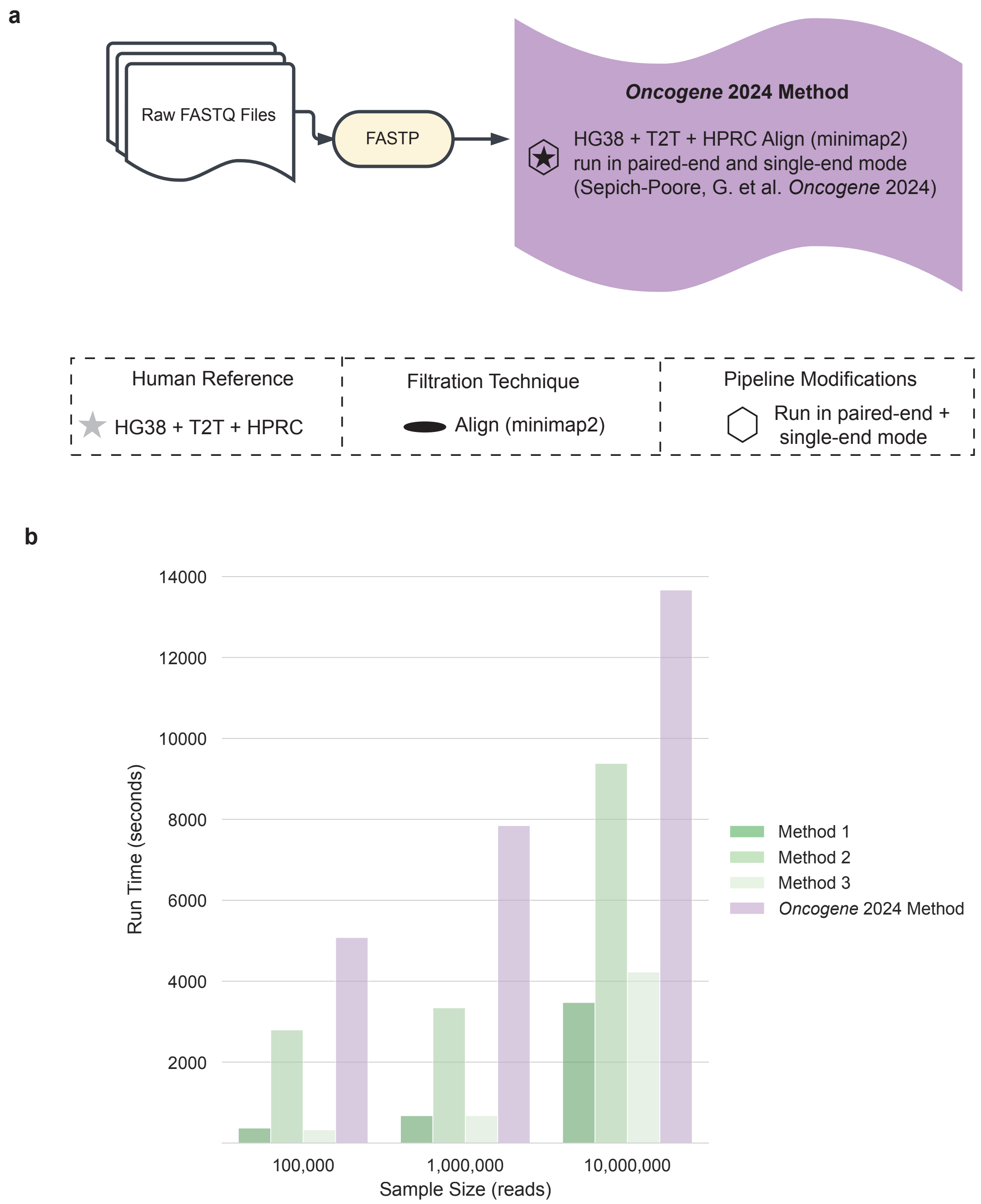

Supplementary Figure 2. Host filtration pipeline runtime evaluation (a) Pipeline of prior host filtration methods (b) Using simulated data with a 50/50 mix of human data from HPRC and microbial data from FDA-ARGOS, we applied the 3 host filtration methods outlined in the manuscript along with the method used in Sepich-Poore, G. et al., *Oncogene* 2024 as outlined in (a) with 3 different sample sizes. Runtimes were averaged across 10 runs per sample size. HG38: GRCH38.p14, T2T: T2T-CHM13v2.0, HPRC: Human Pangenome Reference Consortium 2024 release.

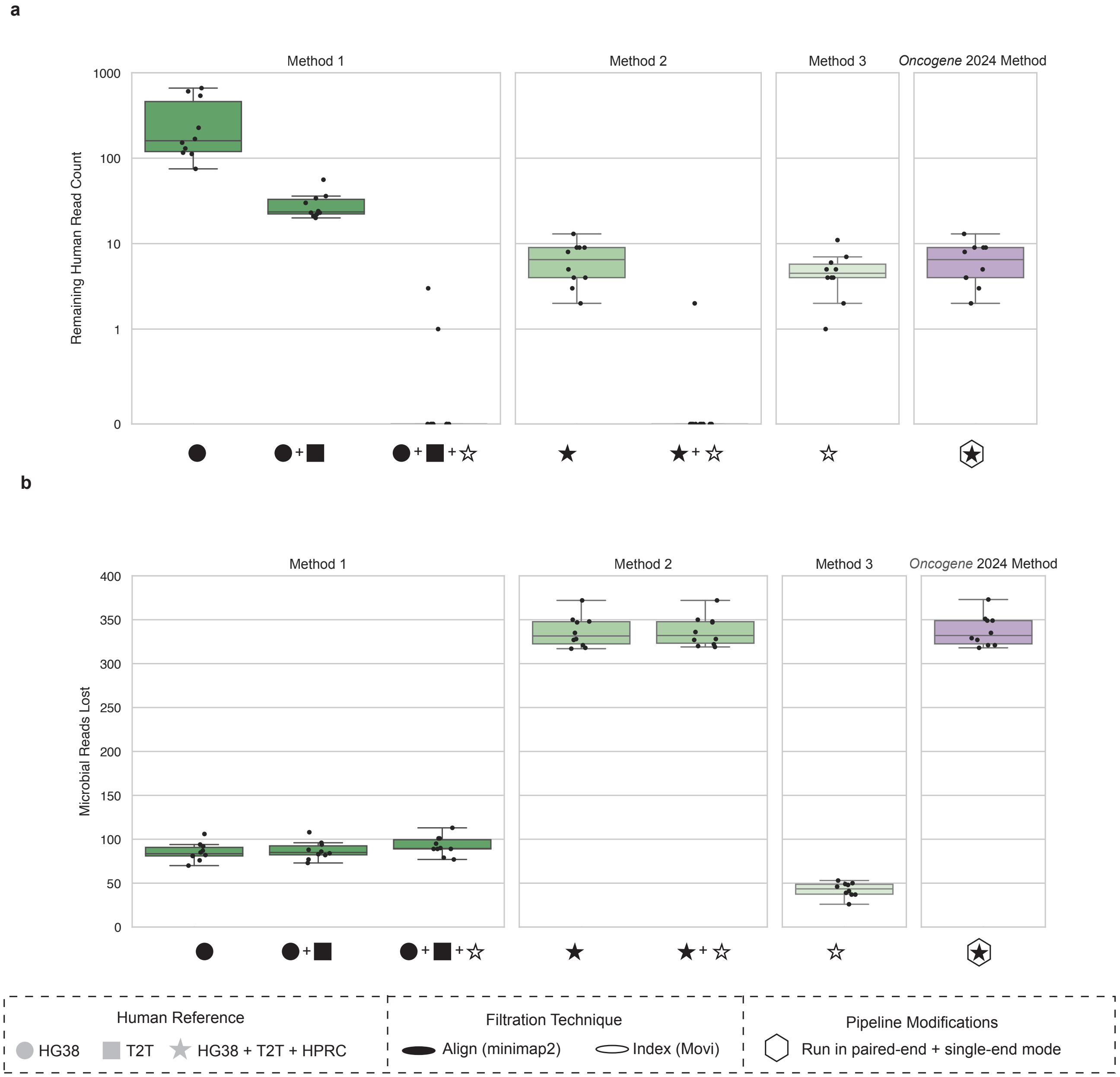

Supplementary Figure 3. Host filtration pipeline simulated data validation Using the 10 simulated datasets of 1 million reads as described in Fig. 2b (a) calculated the number of human reads remaining, and (b) number of microbial reads remaining, for host filtration Methods 1-3 as well as the method used in Sepich-Poore, G. et al., *Oncogene* 2024 which used minimap2 in both paired-end and single-end mode (HPRC host filtration performed excluding the 10 genomes used for simulation). HG38: GRCH38.p14, T2T: T2T-CHM13v2.0, HPRC: Human Pangenome Reference Consortium 2024 release.

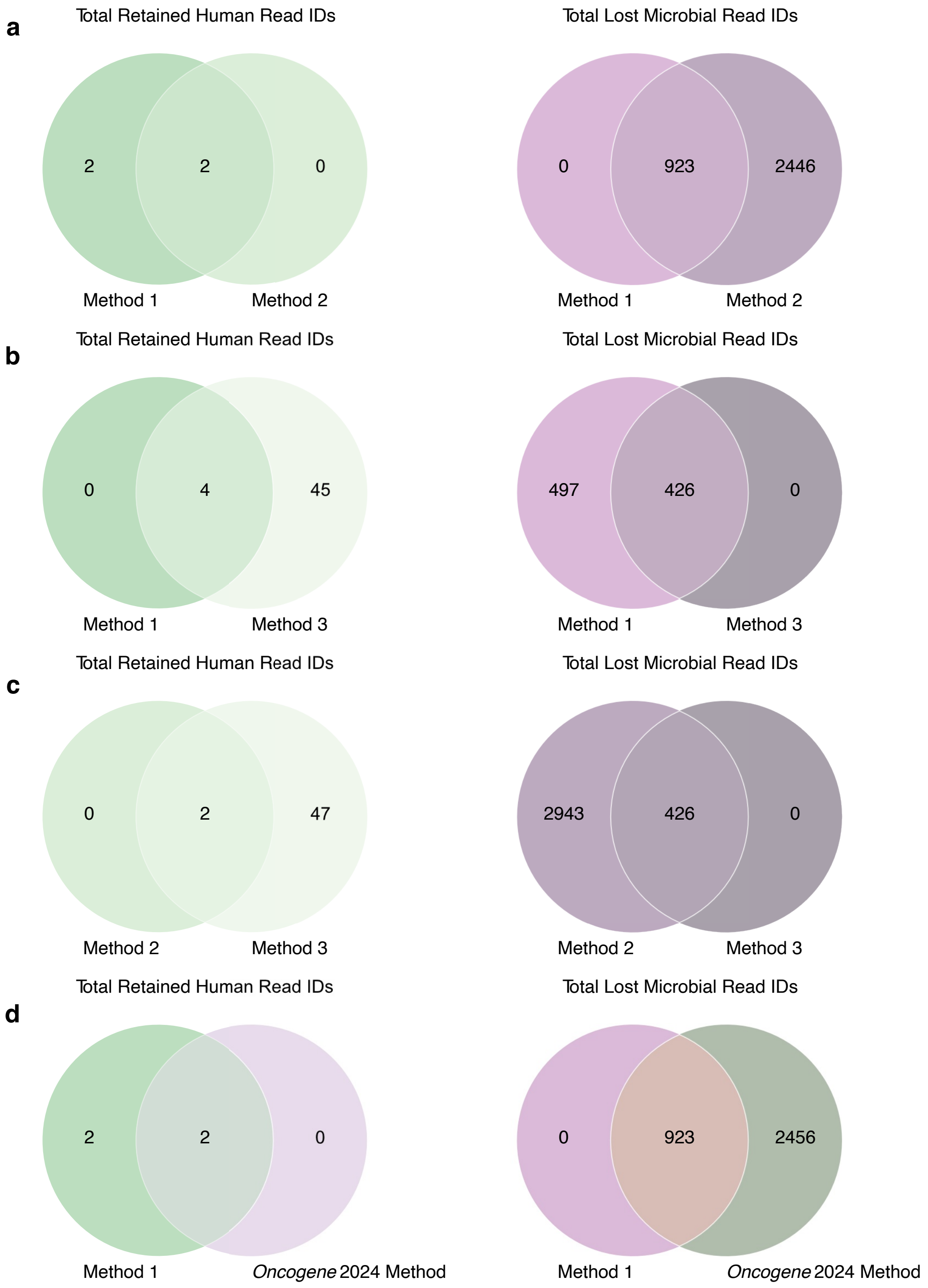

Supplementary Figure 4. Total counts for retained human reads and lost microbial reads of simulated data during benchmarking Using the 10 simulated datasets of 1 million reads as described in Fig. 2b (a) comparison of total unfiltered human reads and total inadvertently filtered microbial reads between Method 1 and Method 2. (b) same as (a) but between Method 1 and Method 3. (c) same as (a) but between Method 2 and Method 3. (d) same as (a) but between Method 1 and the method used in Sepich-Poore, G. et al. *Oncogene* 2024.

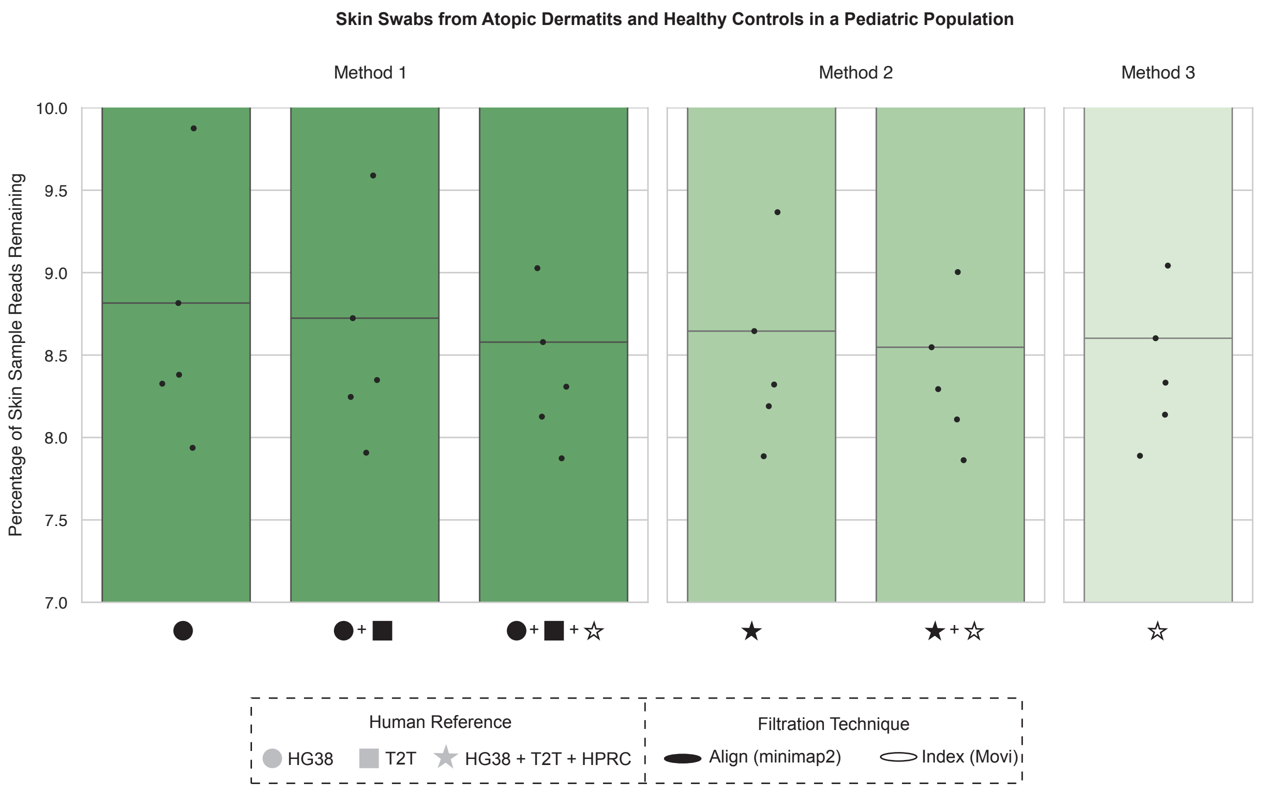

Supplementary Figure 5. Skin Swabs from Atopic Dermatitis and Healthy Controls in Pediatric Population Human skin samples (n=87) were host filtered with the improved methods, we then calculated the percentage of reads remaining for each sample. This figure is identical to Fig. 5a, but zoomed to better view the data. HG38: GRCH38.p14, T2T: T2T-CHM13v2.0, HPRC: Human Pangenome Reference Consortium 2024 release.
