# Supplementary material for: Incomplete human reference genomes can drive false sex biases and expose patient-identifying information in metagenomic data
Source: Nat Commun. 2025 Jan 18;16:825. doi: 10.1038/s41467-025-56077-5 (PMC11742726; doi:10.1038/s41467-025-56077-5)
Supplement: Supplementary file 2 — Description of Additional Supplementary Files [file 41467_2025_56077_MOESM2_ESM.pdf]

## Description of Additional Supplementary Files

**File Name**: Supplementary Data 1
**Description**: Data accessions. This table provides a list of all the datasets used in this study, along with their corresponding accession numbers and repositories.

**File Name**: Supplementary Data 2
**Description**: Impact of various parameters on the identification of sex differences in microbial community composition with and without T2T-CHM13v2.0 filtration. This table provides details on the efficacy of changing various distance metrics, rarefaction levels, and microbial reference databases to resolve the reported artifactual sex effect from HMF.

**File Name**: Supplementary Data 3
**Description**: Taxonomic classification of reads removed by T2T-CHM13v2.0 filtration but retained by GRCh38.p14 filtration. This table presents the results of the taxonomic classification of reads that were removed when filtered with the T2T-CHM13v2.0 reference genome, but retained when filtered with the GRCh38.p14 reference genome. The analysis was performed on a subset of 100 metastatic colorectal tissue samples from HMF. The table lists the taxonomic assignments obtained via Woltka using RefSeq release 200.

**File Name**: Supplementary Data 4
**Description**: Comparison of T2T-filtered read alignment against the original and human-scrubbed microbial databases. This table provides read alignments from reads removed following inclusion of the T2T-CHM13v2.0 human reference genome (Method 1; step ii) onto the human-scrubbed microbial reference database RS210-clean.

**File Name**: Supplementary Data 5
**Description**: Low complexity regions in microbial reference genomes corresponding to coverage peaks of misidentified taxa from T2T-filtered reads. This table details the sequence information of genomic regions containing low complexity sequences in microbial reference genomes from RefSeq release 200. These regions correspond to coverage peaks where reads that were removed following inclusion of the T2T-CHM13v2.0 human reference genome, primarily originating from the human Y chromosome, were incorrectly mapped.

**File Name**: Supplementary Data 6
**Description**: Microbial read count frequencies before and after host filtration across various sample types. This table details the total and median read counts across different sample groups, comparing three different host filtration methods. For each method, the table shows the distribution of read counts before host filtration, after host filtration, and following subsequent alignment to RefSeq release 210. Note, in the case of “Tissue Samples from Various Metastatic Cancers”, the “Before host filtration” count is following GRCh38.p7 host filtration.

**File Name**: Supplementary Data 7

**Description**: False positive taxa identified from insufficient host filtration across various microbial reference databases. This table tabulates the top misidentified taxa computed by aligning reads removed following inclusion of the T2T-CHM13v2.0 human reference genome to various microbial reference genomes and reports the most common organisms.
