# Supplementary material for: Incomplete human reference genomes can drive false sex biases and expose patient-identifying information in metagenomic data
Source: Nat Commun. 2025 Jan 18;16:825. doi: 10.1038/s41467-025-56077-5 (PMC11742726; doi:10.1038/s41467-025-56077-5)
Supplement: Supplementary file 10 — Reporting Summary [file 41467_2025_56077_MOESM10_ESM.pdf]

nature portfolio

Corresponding author(s): Rob Knight

Last updated by author(s): 12/23/2024

Reporting Summary

Nature Portfolio wishes to improve the reproducibility of the work that we publish. This form provides structure for consistency and transparency in reporting. For further information on Nature Portfolio policies, see our Editorial Policies and the Editorial Policy Checklist.

Statistics

For all statistical analyses, confirm that the following items are present in the figure legend, table legend, main text, or Methods section.

n/a	Confirmed	
☐	☒	The exact sample size (*n*) for each experimental group/condition, given as a discrete number and unit of measurement
☐	☒	A statement on whether measurements were taken from distinct samples or whether the same sample was measured repeatedly
☐	☒	The statistical test(s) used AND whether they are one- or two-sided<br>*Only common tests should be described solely by name; describe more complex techniques in the Methods section.*
☒	☐	A description of all covariates tested
☒	☐	A description of any assumptions or corrections, such as tests of normality and adjustment for multiple comparisons
☐	☒	A full description of the statistical parameters including central tendency (e.g. means) or other basic estimates (e.g. regression coefficient) AND variation (e.g. standard deviation) or associated estimates of uncertainty (e.g. confidence intervals)
☐	☒	For null hypothesis testing, the test statistic (e.g. *F*, *t*, *r*) with confidence intervals, effect sizes, degrees of freedom and *P* value noted<br>*Give P values as exact values whenever suitable.*
☒	☐	For Bayesian analysis, information on the choice of priors and Markov chain Monte Carlo settings
☒	☐	For hierarchical and complex designs, identification of the appropriate level for tests and full reporting of outcomes
☒	☐	Estimates of effect sizes (e.g. Cohen's *d*, Pearson's *r*), indicating how they were calculated

Our web collection on statistics for biologists contains articles on many of the points above.

Software and code

Policy information about availability of computer code

Data collection	Data was retrieved from public repositories using wget (v. 1.14) or privately processed using Qiita.
Data analysis	The data in this study was processed using a variety of bioinformatics tools. These tools (in no particular order) include bowtie2 (v. 2.3.5.1), samtools (v. 1.11 and v.1.17), bwa-mem (v. 0.7.x), fastp (v. 0.20.1 and v. 0.23.4), minimap2 (v. 2.17 and v 2.26), Trimmomatic (v 0.39), bcftools (v. 1.10.2), Picard (v. 2.22.8), ART Illumina (v. 2.5.8), QIIME2 (v. 2022.2.0), and Movi (unversioned; git hash 76d5a6da1ec0aeb0121b5ac7c59b295936e23cc1). Custom Python code was developed to facilitate host filtration in this study, and is available publicly (https://github.com/cguccione/human_host_filtration). Jupyter Notebooks were used for figure creation, and these notebooks are available publicly (https://github.com/cguccione/host-filtration-notebooks). Further information on specific utilization of these tools is detailed in the manuscript.

For manuscripts utilizing custom algorithms or software that are central to the research but not yet described in published literature, software must be made available to editors and reviewers. We strongly encourage code deposition in a community repository (e.g. GitHub). See the Nature Portfolio guidelines for submitting code & software for further information.

Data

Policy information about availability of data

All manuscripts must include a data availability statement. This statement should provide the following information, where applicable:

- Accession codes, unique identifiers, or web links for publicly available datasets
- A description of any restrictions on data availability
- For clinical datasets or third party data, please ensure that the statement adheres to our policy

The raw HMF data used in this study is under the purview of the Hartwig Medical Foundation and contains patient-protected information that cannot be shared publicly (see https://hartwigmedical.github.io/documentation/data-access-request-methods.html for data access guidelines for access request details). The FDA-ARGOS database used in this study is publicly available from the official website (https://argos.igs.umaryland.edu) as well as NCBI BioProject PRJNA231221 (https://www.ncbi.nlm.nih.gov/bioproject/231221). The human exome data used in this study is derived from the IGSR phase 3 data, which is available via the official EMBL-EBI portal (https://www.internationalgenome.org/data). The atopic dermatitis skin sample data and the Alzheimer's disease fecal sample data used in this study are available from ENA under accession PRJEB83637 (https://www.ebi.ac.uk/ena/browser/view/PRJEB83637). The fecal sample data from Tomofuji et al. are publicly available from JGA under accessions JGAS000260, JGAS000316, and JGAS000531 (https://www.ddbj.nig.ac.jp/jga/index-e.html). The blood sample data from Tomofuji et al. are publicly available from EGA under accession EGAS00001007027 (https://ega-archive.org/studies/EGAS00001007027).

Research involving human participants, their data, or biological material

Policy information about studies with human participants or human data. See also policy information about sex, gender (identity/presentation), and sexual orientation and race, ethnicity and racism.

Reporting on sex and gender	This study describes a sex-specific artifactual bias we noticed in routine metagenomics analysis and provides readers a means of avoiding such bias in the future. The metagenomics data utilized is derived from human tissue samples, and the sex information is utilized as metadata in routine metagenomics analysis. We do not share any individual level data, nor do we share patient-protected data.
Reporting on race, ethnicity, or other socially relevant groupings	We do not report on race, ethnicity, or other socially relevant groupings.
Population characteristics	We do not report on population characteristics.
Recruitment	We did not recruit participants.
Ethics oversight	This study is retrospective and does not have an ethics board specific to this study.

Note that full information on the approval of the study protocol must also be provided in the manuscript.

Field-specific reporting

Please select the one below that is the best fit for your research. If you are not sure, read the appropriate sections before making your selection.

[x] Life sciences [ ] Behavioural & social sciences [ ] Ecological, evolutionary & environmental sciences

For a reference copy of the document with all sections, see nature.com/documents/nr-reporting-summary-flat.pdf

Life sciences study design

All studies must disclose on these points even when the disclosure is negative.

Sample size	Whenever possible, we utilized all available data from HMF to maximize the sample size. When necessary for computational efficiency, we extracted subsets of the HMF dataset for downstream analysis. Statistical testing was performed (mainly RPCA-PERMANOVA) to validate statistical claims from the aggregate HMF set in the HMF subsets.
Data exclusions	Data exclusion was not relevant to the metagenomics datasets used in the study. We included data from a variety of sample types and across various levels of biomass.
Replication	Replication was performed by leveraging Qiita (study 15693) to run reproducible metagenomics workflows. Reproducibility of the host filtration pipeline is facilitated by the Code Ocean capsule. The aforementioned Qiita studies and EBI links provide access to the host filtered data used in the manuscript analyses.
Randomization	Randomization was not relevant to the metagenomics datasets used in the study as the study explores the technical impact of data preprocessing which is relevant to data generated from mNGS samples.
Blinding	Blinding was not relevant to the metagenomics datasets used in the study as the study explores the technical impact of data preprocessing and does not test a specific experiimental hypothesis that would be impacted by blinding.

Reporting for specific materials, systems and methods

We require information from authors about some types of materials, experimental systems and methods used in many studies. Here, indicate whether each material, system or method listed is relevant to your study. If you are not sure if a list item applies to your research, read the appropriate section before selecting a response.

Materials & experimental systems

n/a	Involved in the study
☒	☐ Antibodies
☒	☐ Eukaryotic cell lines
☒	☐ Palaeontology and archaeology
☒	☐ Animals and other organisms
☒	☐ Clinical data
☒	☐ Dual use research of concern
☒	☐ Plants

Methods

n/a	Involved in the study
☒	☐ ChIP-seq
☒	☐ Flow cytometry
☒	☐ MRI-based neuroimaging

Plants

Seed stocks

Report on the source of all seed stocks or other plant material used. If applicable, state the seed stock centre and catalogue number. If plant specimens were collected from the field, describe the collection location, date and sampling procedures.

Novel plant genotypes

Describe the methods by which all novel plant genotypes were produced. This includes those generated by transgenic approaches, gene editing, chemical/radiation-based mutagenesis and hybridization. For transgenic lines, describe the transformation method, the number of independent lines analyzed and the generation upon which experiments were performed. For gene-edited lines, describe the editor used, the endogenous sequence targeted for editing, the targeting guide RNA sequence (if applicable) and how the editor was applied.

Authentication

Describe any authentication procedures for each seed stock used or novel genotype generated. Describe any experiments used to assess the effect of a mutation and, where applicable, how potential secondary effects (e.g. second site T-DNA insertions, mosiacism, off-target gene editing) were examined.
